# Cetuximab Conjugated with Octreotide and Entrapped Calcium Alginate-beads for Targeting Somatostatin Receptors

**DOI:** 10.1038/s41598-020-61605-y

**Published:** 2020-03-13

**Authors:** Ahmed A. H. Abdellatif, Mohamed A. Ibrahim, Mohammed A. Amin, Hamzah Maswadeh, Muhammed N. Alwehaibi, Sultan N. Al-Harbi, Zayed A. Alharbi, Hamdoon A. Mohammed, Ahmed B. M. Mehany, Imran Saleem

**Affiliations:** 1 Department of Pharmaceutics, College of Pharmacy, Qassim University, Buraydah, 51452 Kingdom of Saudi Arabia; 2 Department of Pharmaceutics and Industrial Pharmacy, Faculty of Pharmacy, Al-Azhar University, Assiut, 71524 Egypt; 3 Kayyali Chair for Pharmaceutical Industries, Department of Pharmaceutics, College of Pharmacy, King Saud University, Riyadh, Saudi Arabia; 4 Pharm. D. Student, College of Pharmacy, Qassim University, Buraydah, 51452 Kingdom of Saudi Arabia; 5 Department of Medicnal Chemistry and Pharmacognosy, College of Pharmacy, Qassim University, Buraydah, 51452 Kingdom of Saudi Arabia; 6 Department of Pharmacognosy, Faculty of Pharmacy, Al-Azhar University, Cairo, Egypt; 7 Department of Zoology, Faculty of Science, Al-Azhar University, Cairo, Egypt; 8 School of Pharmacy & Biomolecular Sciences, Liverpool John Moores University James Parsons Building, Liverpool, UK

**Keywords:** Drug delivery, Nanotechnology in cancer

## Abstract

There is a need to formulate oral cetuximab (CTX) for targeting colorectal cancer, which is reported to express somatostatin receptors (SSTRs). Therefore, coating CTX with a somatostatin analogue such as octreotide (OCT) is beneficial. Alginate was used to coat CTX to facilitate delivery to the gastrointestinal tract (GIT). This study aimed to deliver CTX conjugated with OCT in the form of microparticles as a GIT-targeted SSTR therapy. Both CTX and OCT were conjugated using a solvent evaporation method and the conjugated CTX-OCT was then loaded onto Ca-alginate-beads (CTX-OCT-Alg), which were characterized for drug interactions using differential scanning calorimetry (DSC), and Fourier transform infrared spectra (FTIR). Moreover, the morphology of formulated beads was examined using a scanning electron microscope (SEM). The drug content and release profile were studied using UV spectroscopy. Finally, *in vitro* cytotoxicity of all compounds was evaluated. The results showed homogenous conjugated CTX-OCT with a diameter of 0.4 mm. DSC showed a delay in the OCT peak that appeared after 200 °C due to small polymer interaction that shifted the OCT peak. Moreover, FTIR showed no prominent interaction. SEM showed clear empty cavities in the plain Ca-alginate-beads, while CTX-OCT-Alg showed occupied beads without cavities. CTX-OCT-Alg had a negligible release in 0.1 N HCl, while the CTX-OCT was completely released after 300 min in phosphate buffer pH 7.4. All formulations showed good antiproliferative activity compared with free drugs. The formulated CTX-OCT-Alg are a promising platform for targeting colorectal cancer through GIT.

## Introduction

Oral dosage forms of medications are generally a convenient dosage form for drug delivery^1,2^. However, one of the main challenges in drug delivery is to overcome gastric barriers and preventing the drug release in the stomach^3^. There was a huge increase in the development of orally delivered anti-cancer agents in the past 10 years, as a quarter of all available anti-cancer drugs are now administered orally^4^. Colorectal cancer is one of the most common forms of malignancy and the fourth most common contributor to cancer mortalities^5^. Moreover, targeting specific cells in cancer therapy provides an advantageous way to treat cancer directly^6–10^. For example, the release of drug in the colon and drug particles can be driven by octreotide (OCT) and bind to somatostatin receptors (SSTRs), which are a component of ligand-mediated targeting^8^. The inhibition effect of somatostatin (SST) analogues on tumor cells is one effective approach to cancer therapy. Moreover, SST and its analogues have shown proliferative inhibition of colon cancer cell lines^11^. There are five subtypes of SSTRs expressed in colorectal cancer, and SSTR1 is the most prevalent compared to the others^12^.

Cetuximab (CTX) is a recombinant monoclonal antibody drug used in the management and treatment of colorectal cancer. It is currently only administered intravenously, but there is a need for an oral administered CTX because of the adverse effects such as cardiopulmonary arrest, hypotension, bronchospasm, and dermatological toxicity^13,14^. Cancer development is promoted by EGFR-induced cell growth, migration, and survival^15^. Targeted therapy is a good method for the management of colorectal cancer through the use of “epidermal growth factor receptor inhibitors” (EGFR), such as CTX, which is linked to oral squamous cell carcinoma patient survival^16^. CTX is a chimeric monoclonal antibody that binds to EGFR and inhibits EGFR tyrosine kinase activity, which in turn suppresses EGFR-positive cancer development^17^.

The SSTRs are G-protein-coupled receptors^6–10^, which are expressed in numerous neuroendocrine cancers, including colorectal cancers. They can be targeted with cyclic octapeptides such as octreotide, which is a derivative of native SST. Hence, the conjugation of cargos to octreotides suggests an intelligent approach to cancer treatment^18^. Lelle *et al*. presented a modern approach to drug-carrier conjugate in a site-specific manner providing excellent versatility and allowing stimulated release within cancer cells. They developed doxorubicin-octreotide bioconjugate for overexpressed SSTRs in tumor cells, where the first cleavable disulphide-intercalating connector was the binding between the two elements. The findings demonstrated the delivery platform in biological settings of cytotoxicity studies within cell lines for pancreatic, pituitary, and breast cancer^19^. Hence, selective targeting of the SST receptor expressed on colorectal cancer cells can be achieved by conjugating CTX to OCT, which guides CTX directly to colorectal cancer cells. OCT is a somatostatin analogue commonly used as an anti-cancer agent that targets SSTRs in the GIT that are expressed in colon cancer. However, OCT also has some side effects that are associated with its IV administration, such as local itching at the site of injection^14,20^. Therefore, oral delivery of OCT is an alternative and can enhance the therapeutic activity of CTX orally^21^.

Alginate is naturally found in brown seaweed and can form biodegradable and inert hydrogels that provide safe, orally controlled release^22^. It can deliver drugs and target the colon, and is pH-sensitive, which can aid the controlled release of a drug within a pH 7.4 environment such as Small intestines or colon. Sodium alginate is capable of forming a hydrogel through conjugation with divalent calcium cations (Ca^2+^). Furthermore, the formulation of a drug in the formulated Ca-alginate-beads has many advantages, such as avoiding gastric irritation and providing controlled release of the drug via oral administration due to swelling in the colon rather than in the stomach^23^. Many findings have exhibited the benefit of Ca-alginate-beads in controlling drug release. For example, rifampicin was loaded into Ca-alginate-beads and released in a buffer solution of pH 7.4 but not in a solution of pH 1.2^24^. Another study indicated targeting to the colon through encapsulation of mebendazole using alginate, which provided prolonged colon release of more than 12 hr^25^. Finally, Ca-alginate-beads delivered an anti-cancer agent such as etoposide or that avoided the systemic adverse effects of chemotherapeutic agents and provided targeted, controlled treatment with reduced cytotoxicity^26^.

Colon targeted therapy is widely helpful not only for localized colon cancer treatment but also for the delivery of drugs that are inactivated by acidic pH^2^. Moreover, colonic delivery can be successfully achieved through protecting the drug from gastric release, with a release in the colon at an optimum pH^2,27–29^. However, alginate polymers alone have some limitations, including low mechanical stability, which may facilitate the release and swelling of alginate polymer more rapidly^30^. This study aimed to provide oral colonic targeted therapy using OCT-conjugated CTX to avoid systemic side effects. CTX was conjugated with OCT in Ca-alginate-beads for targeting SSTRs expressed in colorectal cancer. The final formulation was characterized by particle size, DSC, FTIR, *in vitro* release of the formulated CTX-OCT-Alg at phosphate buffer pH 7.4, and cytotoxic activity.

## Results and discussion

### Preparation of the conjugated CTX with OCT

This study aimed to formulate CTX conjugated with OCT in Ca-alginate-beads using sodium alginate polymer for targeting of SSTRs expressed in colorectal cancer. Due to differences in the solubility of OCT and CTX, CTX was coated with OCT forming a soluble CTX-OCT product. OCT is a water-soluble drug, while CTX is soluble in a mixture of methanol and chloroform, forming a clear rather than a cloudy solution. We found one study which used polyethylene glycol (PEG) as a coating polymer for fluconazole in water to exploit the hydrophilicity of PEG; the fluconazole and PEG reaction was carried out by solvent evaporation to form highly soluble fluconazole^28^. The CTX was converted from a water-insoluble material to highly water-soluble CTX-OCT particles that facilitated the transfer of CTX to the receptor site of SSTRs. Moreover, CTX was measured spectrophotometrically at a wavelength of 360 nm, while OCT was measured at 291 nm. It was previously reported that the CTX absorbance could be measured using a multiwall scanning spectrophotometer at 440 nm^31^, while OCT could be detected at 220 nm^32^. The variance between the published wavelength and our method could be due to the difference in the type of instruments used.

**Figure 1:**
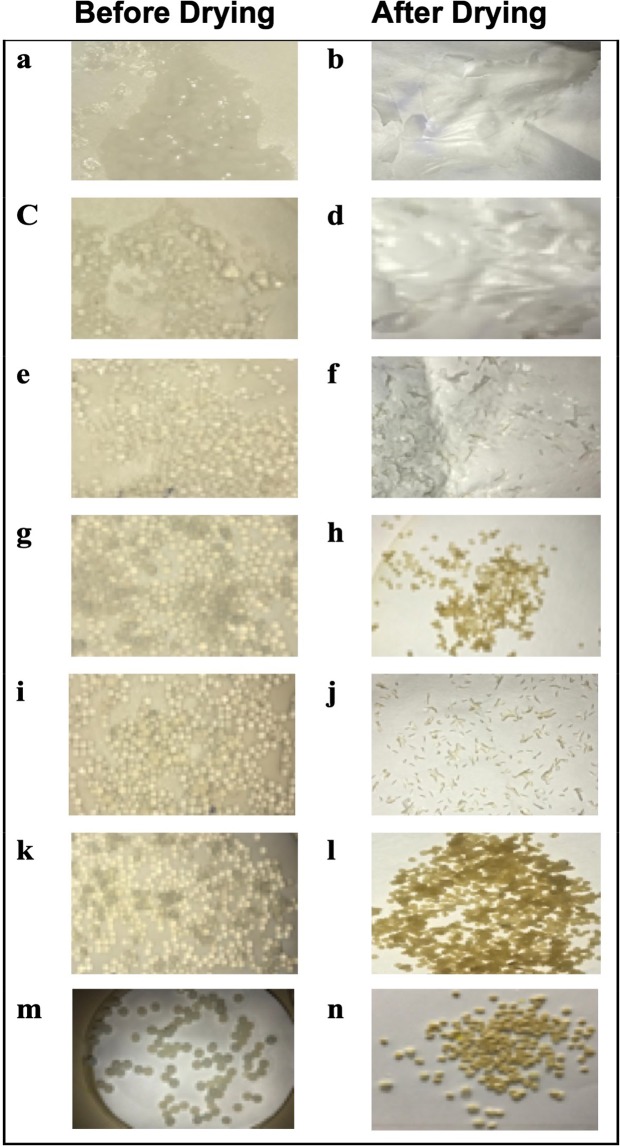
Ca-alginate and CTX-OCT-Alg before and after drying using different concentrations of sodium alginate. (**a**,**b**) plain Ca-alginate-beads (16 µM); (**c**,**d**) dried plain Ca-alginate-beads (22 µM); (**e**,**f**) dried plain Ca-alginate-beads (35 µM); (**g**,**h**) dried plain Ca-alginate-beads (60 µM); (**i**,**j**) dried plain Ca-alginate-beads (82 µM); (**k**,**l**) dried plain Ca-alginate-beads (128 µM; (**m**,**n**) dried CTX-OCT-Alg (128 µM).

The formed Ca-alginate-beads before and after drying were studied at concentrations of 16, 22, 35, 60, and 82 µM, all of which showed uniform beads before drying. However, after drying the beads were irregular in shape and did not hold their form **(**Fig. 1a–j**)**. A concentration of 128 µM (30 mL sodium alginate/10 mL water) produced the most well-formed and stable beads before and after drying (Fig. 1k–n). The formed CTX-OCT particles were loaded into Ca-alginate-beads of uniform shape and size, which could be targeted to the GIT in treatment of colorectal cancer.

### The encapsulation efficiency of the obtained CTX-OCT-Alg

The **encapsulation efficiency** of CTX-OCT in the Ca-alginate-beads ranged between 40–65% for the OCT and between 38–56% for the CTX. Table 1 shows the amount (mg) of CTX and OCT loaded in Ca-alginate-beads. The amount of OCT loaded was 4.5 ± 0.56 mg/10 mg OCT-beads, and 5.9 ± 0.61 mg/10 mg in CTX-OCT-beads, respectively. In addition, the amount of CTX loaded was 6.1 ± 0.91 mg/10 mg CTX-beads and 4.1 ± 0.34 mg/10 mg in CTX-OCT-Alg, respectively. The amount of CTX and OCT loaded into CTX-OCT-Alg were reasonable as the amount initially used in the formulation was considered to be the saturated solution of CTX-OCT^33^.

**Table 1:** Amount of drug loaded into Ca-alginate-beads measured using the wavelengths of octreotide and cetuximab.

Beads Sample	Amount of drug (mg) in 10 mg beads ± SD	
	Octreotide (OCT)	Cetuximab (CTX)
Octreotide-beads	4.5 ± 0.56	—
Cetuximab-beads	—	6.1 ± 0.91
Cetuximab-octreotide-beads	5.9 ± 0.61	4.1 ± 0.34

### Size, morphology and swelling determination

**Figure 2:**
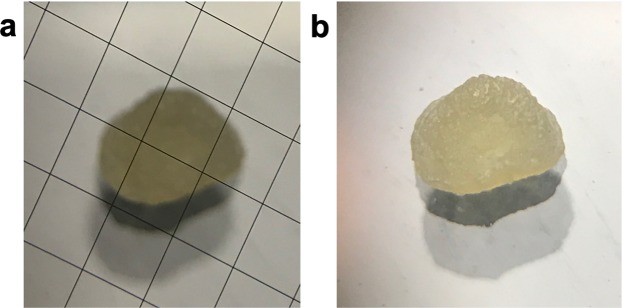
Cetuximab-Octreotide alginate bead image using an Ultracut UCT Ultra-microtome (**a**) Cetuximab-octreotide bead with visible measurement box markings (**b**) Cetuximab-octreotide bead without visible metered boxes.

The formed beads at a concentration of 128 µM alginate were characterized to determine bead size. The prepared beads were measured at a resolution of 1.5× to determine the diameter of beads on a slide marked with 100-µm squares. The formulated beads were equal to four squares in width and height, with a diameter of 0.4 mm (Fig. 2**)**, which was calculated as 4 squares × 100 µm = 400 µm. Another study found that formulated Ca-alginate-beads had diameters that ranged from 700 to 1400 μm^34^. This difference in size was due to the different formulation methods, in this study alginate was dropped into the CaCl_2_ solution, whilst the grinded beads showed ranged size of 1–2 µm.

**Figure 3:**
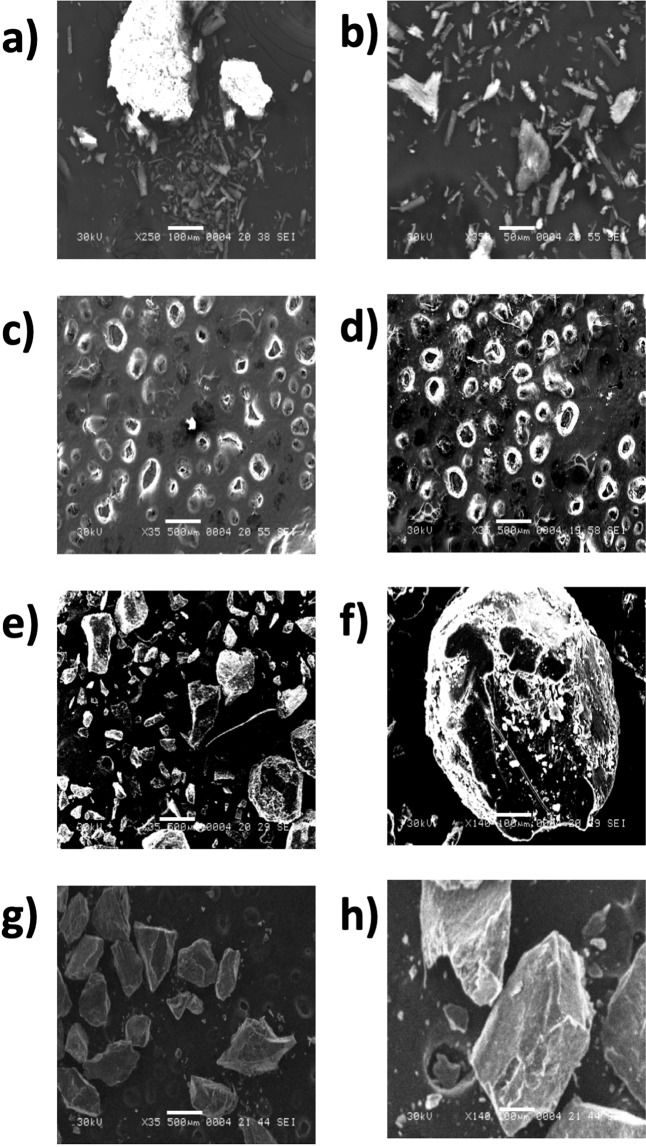
Scanning Electron Microscopy of (**a**,**b**) cetuximab alone, (**c,d**) cetuximab-octreotide conjugation, (**e**) Plain beads at 35×, 500 µm (**f**) 140×, 100 µm, (**g**) cetuximab-octreotide beads at 35 × 500 µm, and (**h**) cetuximab-octreotide beads at 35 × 500 µm.

CTX free drug was imaged using SEM and observed to be composed of heterogeneous particles (Fig. 3a,b). However, after conjugation of CTX to OCT, the particles were homogenous (Fig. 3c,d), which confirmed the coating of CTX with OCT. Moreover, plain beads were imaged using at magnifications 35×, 500 µm and 140×, 100 µm. In these images, the powders had empty cavities (Fig. 3e,f). In contrast, Ca-alginate-beads loaded with CTX-OCT powders had turbid and fill cavities, which indicated the physical presence of drug powder (Fig. 3g,h). Another study that used SEM to evaluate alginate bead shape found an irregular shape of sodium alginate before coating, that resolved to a homogenous and uniform shape after coating^28^, which was consistent with our findings (Fig. 3). This indicates that CTX alone does not confer irregular alginate bead structure, but after its conjugation with OCT, the beads take on a uniform and homogenous shape, which serves as an indication that OCT coating of CTX results in uniform structures.

**Figure 4:**
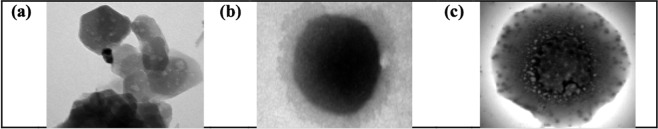
Transmission Electron Microscope (**a**) the plain cetuximab, (**b**) cetuximab coated octreotide, and (**c**) cetuximab-octreotide incorporated beads. Scale bar is 500 µm.

TEM evaluation was used to examine the internal structure and morphology of CTX-OCT-Alg. The beads before milling exhibited irregular shapes with particle sizes <0.4 mm (Fig. 4). The internal structure of the CTX particles had no core (i.e. drug loading/**encapsulation efficiency**) and therefore, no distinct shape (Fig. 4a). After the CTX was coated with a layer of the stabilizing OCT, the particles were stable and soluble, and the CTX had a visible coating layer of OCT (Fig. 4b). TEM of CTX-OCT-Alg confirmed that CTX-OCT formed complexes and were incorporated inside the beads (Fig. 4c). Similar results were reported by Page *et al*., who showed vesicle-like shapes inside beads incorporated drugs^35^.

### Entrapment and drug interaction of CTX-OCT in Ca-alginate-beads

**Figure 5:**
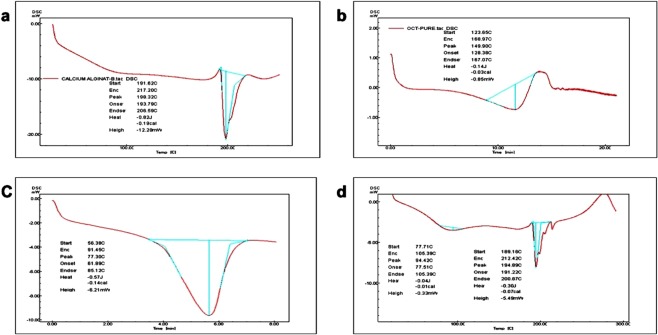
Differential scanning calorimetry of (**a**) Ca-alginate-beads, (**b**) octreotide, (**c**) cetuximab, (**d**) cetuximab-octreotide-alginate-beads.

The thermal analysis of sodium alginate showed a broad endothermic peak that began at 145.5 °C, which indicated that the compound was pure. Moreover, the same peak was observed in the physical mixture of CTX-OCT (Fig. 5d). Pure OCT powder produced an endothermic peak at 123 °C that ended at 168 °C, which represented the melting point of OCT (Fig. 5b). The pure CTX drug had an endothermic peak that began at 56 °C and it ended at 91.4 °C; these peaks indicated the melting point of CTX (Fig. 5c). We then analyzed Ca-alginate-beads and observed approximately the same endothermic peak as sodium alginate **(**Fig. 5a**)**, which indicated no interaction between CTX-OCT conjugate and sodium alginate and indicate the CTX-OCT can be released from Ca-alginate-beads. DSC analysis of CTX-OCT-Alg showed endothermic peaks at 77.71 °C that represented CTX and a delay in the OCT peak that appeared after 200 °C, indicating a slight polymer interaction that shifted the OCT peak from 150 °C to 200 °C, this predicted reaction led us for formulate CTX-OCT (Fig. 5d).

**Figure 6:**
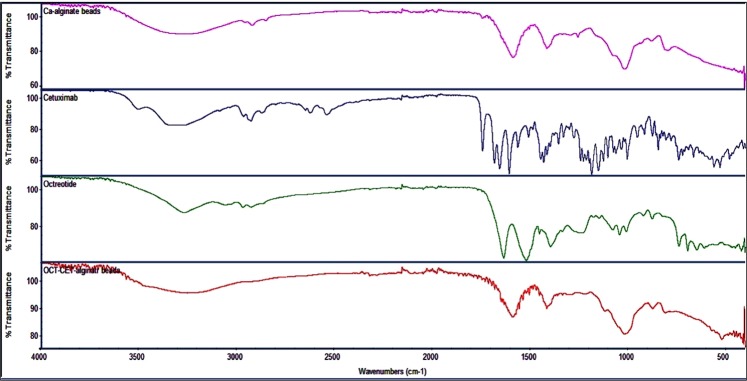
Cetuximab-octreotide raw powder and beads powder analysis through Fourier-transform infrared spectroscopy.

To examine the drug interaction between OCT and CTX, FTIR was used. Absorption bands showed no prominent interaction between the two compounds, and CTX-OCT bead powder had similar absorption bands as their raw powders. Bands between 3000–3600 cm^−1^ indicated O-H stretching in alginic acid, and OH in OCT. Bands between 1670 and 1460 cm^−1^ were related to asymmetrical and symmetrical stretching vibrations of carboxylate salt ions in alginate and OCT. C-H stretching was presented at a range of 2955–2870 cm^−1^, while C-O stretching of the pyranose ring in alginate and OCT appeared at 1100 cm^−1^. Ca-alginate had a narrow O-H band compared to sodium alginate due to a decrease in OH groups from Ca-alginate chelation. The Ca ion substitution of the sodium ion resulted in a change in atomic weight and charge density; thus, asymmetric stretching vibrations were shifted to a lower wavelength (Fig. 6**)**. Moreover, the FTIR studies showed no interaction between the CTX, OCT, and alginate, which was consistent with other results that used FTIR to confirm the physical interaction of all components but found no interaction between CTX, OCT and alginate^32,36^.

Alginic acid is an anionic polymer, hence the carboxyl groups are not ionized at a pH lower than the pKa, swelling of Ca-alginate beads is negligible under an acidic condition. Any product release thus mostly is due to the distribution of the medication over the insoluble matrix. Nevertheless the anionic polymer is more ionized and swells under neutral and basic conditions after which drug release is dependent on the beads’ swelling^37^. Bead swelling percent in HCl showed that very low change in diameter, which indicated a very low swelling percentage. In contrast, beads in phosphate buffer showed a gradual increase in diameter size, indicating a high swelling percentage (Table 2).

**Table 2:** Diameter size differences between plain beads dissolved in HCl and phosphate buffer, pH 7.4.

Time (min)	Bead Diameter in HCl pH 1.2 (mm)	Bead Diameter in phosphate buffer pH 7.4 (mm)
0	0.4	0.4
30	0.41	0.45
60	0.41	0.45
90	0.42	0.55
120	0.43	0.60
150	0.44	0.65
180	0.44	0.70
210	0.45	0.80
240	0.45	Swell and burst

### Release of CTX-OCT from Ca-alginate-beads

**Figure 7:**
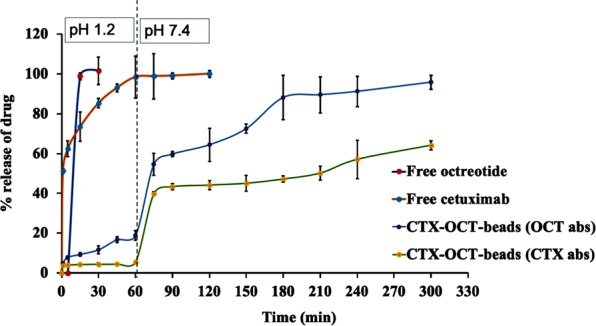
*In vitro* release of cetuximab-octreotide beads and raw powders. Free octreotide (blue line), free cetuximab (red line), cetuximab-octreotide according to octreotide (green line), and cetuximab-octreotide according to cetuximab (orange line). The results were expressed as the mean standard deviation of triplicate data in a single experiment.

The release of CTX-OCT from Ca-alginate-beads relied on the diffusion of the release medium into the Ca-alginate-beads, swelling and dissolution of the alginate matrix. During the first 60 min in acidic media, none of the formulations released the drug compound. After this initial time, the release rates between the formulations were statistically significant (p < 0.05) in alkaline media, pH 7.4 (Fig. 7). In the alkaline medium, the release of both OCT and CTX was biphasic, in which an initial burst was detected after pH changed, followed by a sustained release pattern. During the first stage after pH change (60–90 min), two superficial mechanisms, swelling and diffusion, control drug release from the beads. During the second phase from 90–300 min, the enlargement of the beads was continuous while did not affect drug release. Comparing the drug release from free and loaded beads, the alginate significantly reduced the release profile of the drug from beads. These results show that the free drugs were completely dissolved in the first 30 min in acidic pH, which justifies the role of alginate as a coat that can significantly increase the targeting and release of drug in GIT.

Once exposed to acidic media, Ca-alginate-beads tend to shrink. The carboxylates of the Ca-alginate-beads are protonated at low pH values (<4), which decreases and shrinks the electrostatic repulsion between these groups^38,39^. In addition, the swelling/bruising curve begins to decline in the somewhat basic PBS environment, implying dissolution or decay^40^. Additional, swelling/bruising of the dried beads was typically due to the hydration of the hydrophilic groups of alginate^41^. In this case, free water penetrates the beads and thus promotes a bigger swelling level, filling the inert pores among the polymer chains while no swelling was observed in acidic media. In the mild alkaline environment of PBS, all preparatory products showed significant swelling levels. In this case, the swelling was associated with the exchange of Ca2+ and ion Na+.^40^.

CTX-OCT’s alginate-bead release profile of 0.1 N HCl, pH 1.2 is shown in **(**Fig. 7**)**, with 5.5% and 18.8% of the drug content discharged, in CTX-loaded and OCT-loaded-beads, respectively after 1 hr. The OCT powder in the acidic medium in comparison showed full release of the drug content after 1 hr. The highest peak of CTX release after 300 minutes was 64.2% and 95.7% of OCT. Zero-Order, First Order, Hixson–Crowell and Higuchi equations were used for the analysis of the separation kinetics and the process of the liberation of drugs from prepared Ca-alginate-beads. Table 3 shows that the release kinetics were better suited to the first-order equation and that the release rates were dependent on concentration. Furthermore, the mechanism by which OCT and CTX were released from the Ca-alginate-beads produced in this study was best suited to the Hixson-Crownell equation. Previous studies reported that the release of drugs from Ca-alginate-beads was best fit to the Higuchi equation^42,43^, which characterized the mechanism of release as diffusion-controlled.

**Table 3:** Rate constants and correlation coefficients for all formulations using zero-order, first-order, Higuchi, and Hixson-Crowell equations.

Brand	Zero-order dissolution rate constant	First-order dissolution rate constant	Higuchi dissolution rate constant	Hixson-Crowell dissolution rate constant
**Free OCT**				
Free CTX	0.428 r^2^ = 0.826	0.0397 r^2^ = 0.933	5.153 r^2^ = 0.914	0.428 r^2^ = 0.976
CTX-OCT-beads (OCT abs)	0.352 r^2^ = 0.904	0.009 r^2^ = 0.949	6.806 r^2^ = 0.918	0.244 r^2^ = 0.934
CTX-OCT-beads (CTX abs)	0.215 r^2^ = 0.808	0.003 r^2^ = 0.850	4.312 r^2^ = 0.848	0.106 r^2^ = 0.891

Free OCT = free octreotide, Free CTX = free cetuximab, CTX-OCT-beads (OCT abs) = cetuximab-octreotide beads depending on the absorbance of octreotide, CTX-OCT-beads (CTX abs) = cetuximab-octreotide beads depending on the absorbance of cetuximab.

One of the limitations of formulating CTX-OCT in Ca-alginate-beads is the low mechanical stability of the beads, which may enhance the swelling of the alginate polymer and accelerate drug release. Coating Ca-alginate-beads with cationic polymers can overcome this problem low mechanical stability. Our *in vitro* release results showed that CTX-OCT-Alg have a low drug release rate after 60 min in 0.1 HCl 1.2 pH, while in phosphate buffer pH 7.4, all drug contents were released after 300 min. Results from flurbiprofen loaded Ca-alginate showed less than 5% of the drug content was released after 1 hr^23^, which support our finding that CTX release in 0.1 HCL 1.2 pH was less than 5% of drug content released after 1 h; however, OCT drug released was higher but still less than 20%. CTX-OCT-Alg in phosphate buffer 7.4 revealed a gradual increase of the drug release rate until complete release after 5 h. This finding agrees with the data from theophylline coated by alginate for colon targeting delivery, which had complete drug release after 7 h. This delay in release was attributed to the coating of Ca-alginate-beads with another polymer such as chitosan, which provided more release control compared to our results with CTX-OCT-loaded beads^44^.

**Figure 8:**
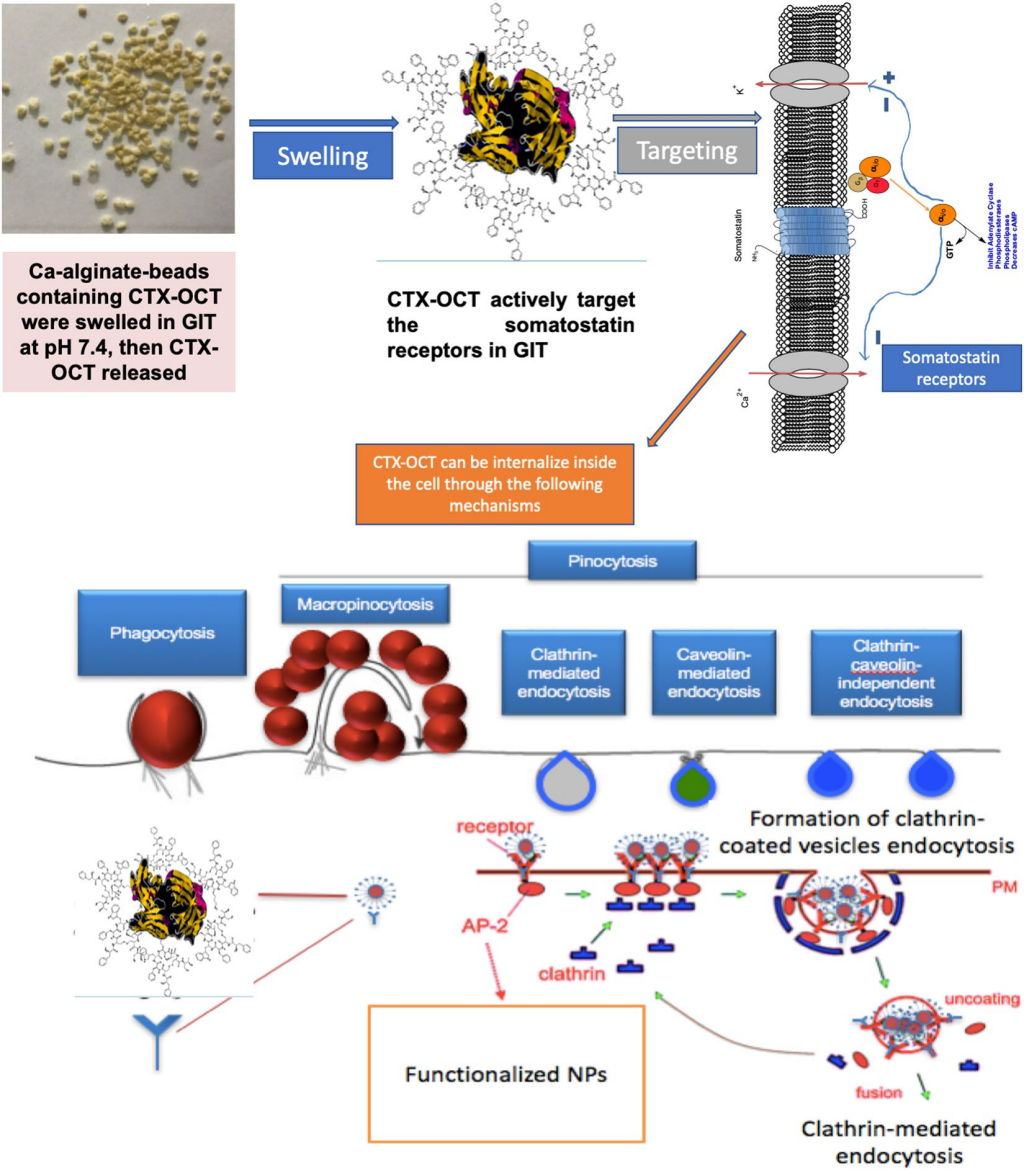
Mechanistic diagram of the CTX-OCT release from Ca-alginate-beads and target the somatostatin receptor.

The mechanistic pathway of Ca-alginate-beads containing CTX-OCT can interpreted by swelling of Ca-alginate-beads then releasing of CTX-OCT in GIT medium. The CTX-OCT particles can be uptake through receptor activation within a mammalian cell. CTX-OCT can be internalize through endocytic vesicles by both phagocytosis and pinocytosis pathways^45^. Phagocytes are the more favored mechanism which can take up large particles (also, aggregates), or particles with certain ligand such as OCT. Moreover, the non-accepted mechanism for particles internalization in a non-phagocytic mammalian cell is commonly throughout pinocytosis or direct diffusion. Furthermore, particles can be taken up via specific internalization (receptor-mediated) endocytosis or nonspecific endocytosis^45^. The receptor-mediated endocytosis authorizes an introduction of extracellular big molecules (Fig. 8)^46,47^. In this phase, complex membrane micro-domains containing clathrins or caveolin-coated pits shape the plasma membrane^47^. Nevertheless, the precise absorption of nanoparticles through means of surface receptors can be improved either through direct interactions between coated particles and receptors or via nanoparticles ligands^47–49^.

### Determination of the antiproliferative activity of CTX-OCT formulations

The effects of tested compounds on human cancer cell lines HCT-116, HepG-2, and MCF-7 were measured and recorded in Table 4. The viability of the cells treated with OCT-alginate and CTX-alginate-beads significantly decreased compared to the free OCT and CTX treatments. This result could be due to the improved solubility of the OCT-alginate and CTX-alginate-beads, which improved cell penetration for both OCT and CTX in all cell lines as the antiproliferative activity for both OCT and CTX were improved^50,51^. For example, the HCT-116 cell line had an IC_50_ for OCT-alginate and CTX-alginate-beads of 5.48 µg/mL and 4.38 µg/mL vs 7.92 µg/mL and 5.21 µg/mL for free OCT and CTX, respectively. In addition, HEPG-2 cells showed a distinct reduction in the IC_50_ of OCT and CTX-alginate-beads (IC_50_ = 5.76 µg/mL and 3.82 µg/mL, respectively) compared to free OCT and CTX (IC_50_ = 10.26 µg/mL and 7.32 µg/mL, respectively). However, the outcomes exhibited less cytotoxic activity for CTX-OCT-Alginate-beads paralleled to the OCT-alginate or CTX-alginate-beads in all the tested cell lines^50,51^. These results suggest the coating of CTX with OCT decreased CTX activity by making a barrier layer around the molecules. Furthermore, CTX-OCT-Alginate-beads had the same activity as free CTX and were more active than free OCT. These findings indicate that the Ca-alginate-beads loaded with CTX-OCT can target the specific area in GIT at pH 7.4, allowing CTX-OCT to enter cells that express the SSTRs and then dissociate into OCT and CTX with anti-cancer activity. Importantly, the activities of all formulations were synergistic or similar to the free drugs, indicating that the anti-cancer activity was not affected by the alginate bead coating.

**Table 4:** Antiproliferative activity of octreotide, cetuximab, and their formulated Ca-alginate-beads.

No.	Code	IC_50_ (µg/mL)		
		HCT-116	HEPG-2	MCF-7
1	Calcium alginate-beads	30.5 ± 2.1	26.7 ± 1.9	32.8 ± 3.1
2	Free OCT	7.92 ± 0.61	10.26 ± 0.97	12.61 ± 0.98
3	Free CTX	5.21 ± 0.44	7.32 ± 0.58	8.59 ± 0.78
4	OCT-alginate-beads	5.48 ± 0.47	5.76 ± 0.48	7.09 ± 0.55
5	CTX-alginate-beads	4.38 ± 0.38	3.82 ± 0.24	5.17 ± 0.42
6	CTX-OCT-Alginate-beads	6.27 ± 0.56	8.13 ± 0.73	8.54 ± 0.76

Free OCT = free octreotide, Free CTX = free cetuximab, OCT-alginate-beads = octreotide alginate-beads, CTX-alginate-beads = cetuximab alginate-beads, CTX-OCT-beads = cetuximab-octreotide beads.

## Materials and Methods

### Materials

Cetuximab and OCT were purchased from Hong Kong Yuancheng Saichuang Technology Co. Limited (Hong Kong, China). Alginic acid sodium salt high viscosity grade A was purchased from MP Biomedicals (Eschwege, Germany). Calcium chloride (CaCl_2_), 99.5% methanol, hydrochloric acid, and dibasic sodium phosphate heptahydrate were purchased from Loba Chemie Pve. (Mumbai, India). Chloroform was purchased from Techno Pharmchem (Haryana, India). The mammary gland breast cancer cell line (MCF-7), human hepatocellular carcinoma cell line (HepG-2), and colon carcinoma cell line (HCT-116) were obtained from VACSERA-Cell Culture Unit (Cairo, Egypt). RPMI-1640 medium, SRB (SulphoRhodamine-B), DMSO (dimethyl sulfoxide), and doxorubicin were purchased from Sigma Co. (St. Louis, USA). Fetal bovine serum was obtained from Sigma Aldrich (Irvine, UK). All chemicals used in this study were of analytical grade and used as received without any further purification. All prepared solutions were prepared with deionized water.

### Methodology

#### Preparation of the conjugated CTX-OCT-Alg

For preparing of Ca-alginate-beads loaded with CTX-OCT. A stock solution of sodium alginate was prepared by dissolving 2 g sodium alginate powder in 100 mL distilled water with the aid of magnetic stirring at 100 rpm for 1 hr^52^. Six diluted concentrations of sodium alginate were prepared at 16 µM, 22 µM, 35 µM, 60 µM, 82 µM, and 128 µM. For preparing of calcium chloride solution, one hundred milliliters 0.2 M CaCl_2_ were prepared by adding 2.22 g to 100 mL water, which was then mixed by the aid of magnetic stirring at 100 rpm for 1 min^52^. Moreover, sodium alginate solution was dropped into CaCl_2_. Each concentration of sodium alginate was dropped into (0.2 M) calcium chloride solution using a 2-mL pipette and mixed with the aid of magnetic stirring at 100 rpm.

**Figure 9:**
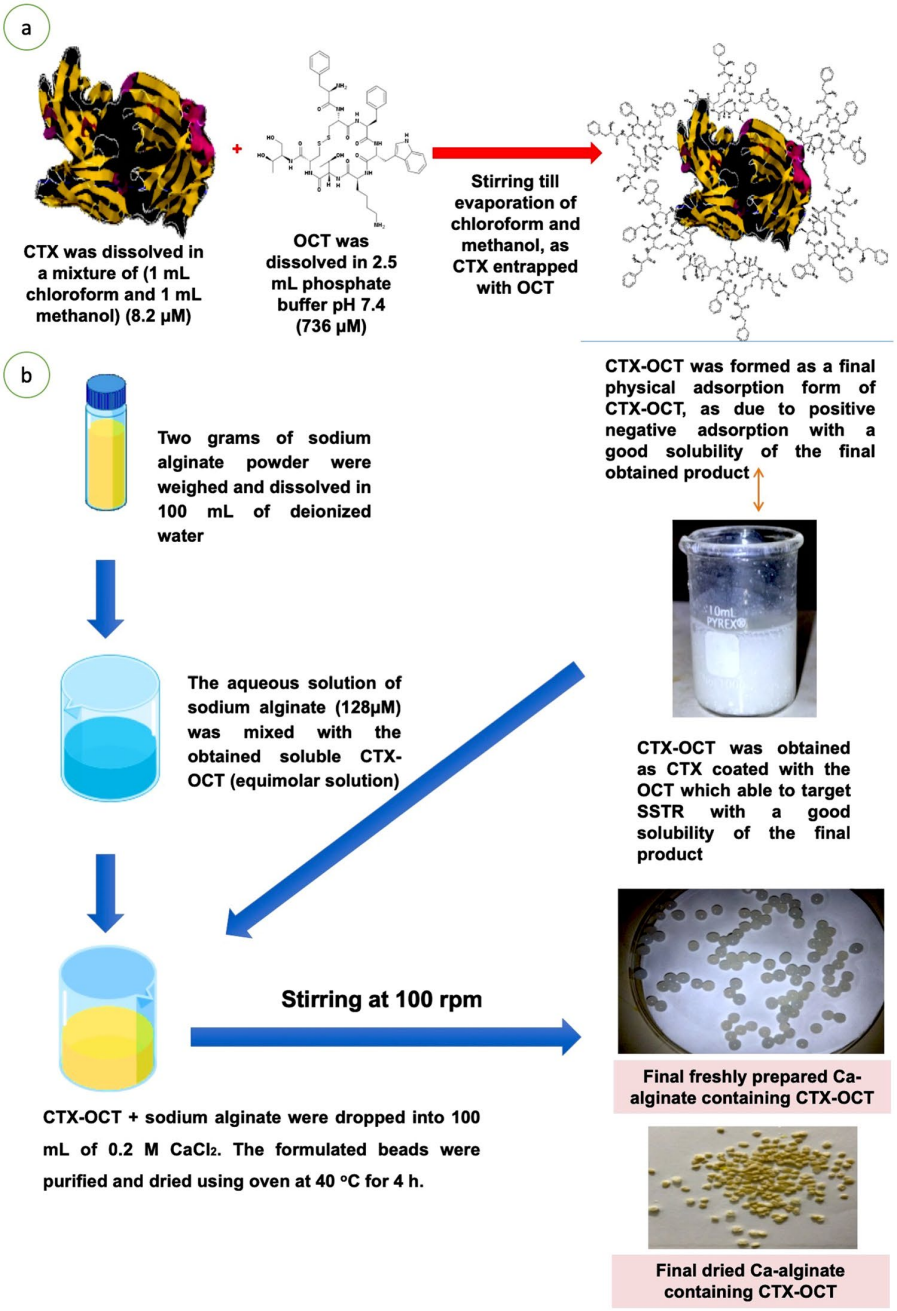
(**a**) Physical adsorption of octreotide to cetuximab depends on their different solubilities in aqueous media. (**b**) Formation of Ca-alginate-beads loaded with conjugated of cetuximab-octreotide.

The coating of OCT to CTX depends on their solubility difference in aqueous and organic media; CTX can be coated with OCT using the solvent evaporation method^53^ for SSRT targeting. OCT is a water-soluble drug, while CTX is solubilized in 1 mL 1:1 methanol and chloroform mixture. Briefly, each drug was separately mixed with a suitable solvent; 30 mg OCT was dissolved in 2.5 mL phosphate buffer and 50 mg CTX was dissolved in 1 mL 1:1 methanol and chloroform. The mixture was left to mix for 4 hr at 100 rpm to evaporate the methanol and chloroform and facilitate the OCT coating of CTX. The reaction produced 8.2 µM CTX that was soluble in water due to being coated with 736 µM OCT (Fig. 9).

### Preparation of CTX-OCT-loaded Ca-alginate-beads

The conjugated CTX-OCT solution was mixed with 128 µM sodium alginate and then dropped into 100 ml 0.2 M CaCl_2_ using a 2-mL pipette, and stirred at 100 rpm. The obtained beads were loaded with CTX-OCT. The formulated beads were purified by filtration through 90-mm filter paper obtained from Sigma Aldrich (Irvine, UK). Finally, formulated beads were dried in an oven at 40 °C for 4 hr (Fig. 9).

### Determination of encapsulation efficiency

To determine the drug content/encapsulation efficiency of the CTX-OCT-Alg, 10 mg beads were placed in 100 mL phosphate buffer, pH 7.4. The mixture was stirred for 6 hr until all beads dissolved and released the CTX-OCT content. The solution was measured for encapsulation efficiency of CTX-OCT using a UV spectrophotometer (Varian Cary® 50 UV-Vis Spectrophotometer, Port Melbourne, Australia) at a wavelength of 360 nm, while OCT was measured at 291 nm. Drug content was computed using a calibration curve (R^2^ = 0.9998) prepared from CTX-OCT solutions with concentrations ranging 1–6 µg/mL. The drug loading capacity of the Ca-alginate-beads was calculated according to the following equation:

$${\rm{Drug}}\,{\rm{Loading}}\,( \% )=\frac{(Total\,amount\,of\,drug\,in\,particle)}{weight\,of\,particles\,taken}\times 100$$

The actual concentration of obtained CTX-OCT-Alg was determined by a comparison between the concentration of a known standard and the concentration of unknown sample using different diluted concentrations of both CTX and OCT^54^.

### Size measurement

The size of CTX-OCT-Alg were assessed with an optical microscope fitted with a stage micrometer using a Leica Ultracut UCT Ultra-microtome (Mannheim, Germany) with the accuracy of 0.5–3 mm. Analysis of prepared beads was performed at a resolution of 1.5× to determine the diameter of beads with each square equal to 100, and then calculated bead diameter by the number of squares occupied^55^.

### Scanning electron microscopy (SEM)

Five milligrams of each empty Ca-alginate and CTX-OCT-Alg were milled into a fine powder with a mortar and pestle. SEM was carried out using a JEOL JSM-550 Scanning Electron Microscope (Jeol, Akishima, Tokyo, Japan) to analyze the superficial morphology of the formulated bead powder. Randomly chosen bead powder samples were placed on the surface double-sided copper conductive tape. The beads were then sputter-coated (Sputter coater, JOEL JFC-1300) with a thin layer of platinum in a vacuum for 55 s at 25 mÅ using a coating unit to make it electrically conductive before imaging in an SEM instrument^56^.

### Transmission electron microscopy (TEM)

A transmission electron microscope (TEM) was used to characterize the exterior and interior shape of Ca-alginate-beads particles (FEI Tecnai G2 20 TWIN, USA) that was operated at 200 kV. The point-to-point with the resolution of this microscope was less than 0.25 nm, while the line-to-line resolution was less than 0.10 nm^57^.

### Differential scanning calorimetry (DSC)

To study the thermal behavior of CTX, OCT, CTX-OCT, and CTX-OCT-Alg, DSC measurements were conducted using a DSC-60 calorimeter (Shimadzu, Kyoto, Japan)^58^. DSC-60 was used for thermal analysis of CTX-OCT-Alg and their powders, sodium alginate powder and ca-alginate-beads. Exactly 5 mg of CTX-OCT-Alg were placed on aluminum pans and assessed for 27 min at 10 °C per min, and then 5 mg of each raw drug powder was assessed separately under same conditions. The DSC thermogram of conjugated CTX-OCT, sodium alginate and CTX-OCT powder was analyzed to confirm the materials were pure, confirm conjugation, and to identify any drug-polymer interactions.

### Fourier-transform infrared spectroscopy (FTIR)

FTIR was used to test the compatibility of all components together; therefore, we aimed to detect any drug interaction between the drug and polymer by collecting between 400 and 4000 cm^−1^. CTX-OCT loaded alginate-bead powder, sodium alginate, OCT powder, and CTX powder were subjected to FTIR (Nicolet™ iS50 FTIR Spectrometer, Thermo SCIENTIFIC Co., Twin, USA)^32,36^.

### Swelling percentage

Percentage of swelling was studied by investigating the penetration of HCl or phosphate buffer into CTX-OCT-Alg^55^. Eight beads were tested for their swelling in 50 mL 0.1 HCl for 1 hr followed by 50 mL phosphate buffer for another 3 h. The swelling % was determined and presented as a graph.

### *In vitro* release of CTX-OCT-Alg

In a dissolution tester (Validata–Hansen study Chatsworth, Ca, USA), a aliquot of CTX-OCT loaded Ca-alginate-beads equal to 50 mg per medication rotated at 50 rpm. 1 mL sample was withdrawn and substituted by 1 mL of phosphate buffer at predefined times. The CTX and OCT published rates were measured using UV spectroscopy for 360 nm for CTX and 292 nm for OCT, depending on the calibration curve^23^. The CTX-OCT-Alg were placed in 50 mL 0.1 N HCl (pH 1.2) for 60 min, then the same beads were filtered and retested in phosphate buffer (pH 7.4) for an additional 210 min. The released CTX-OCT from the beads was analyzed spectrophotometrically at a wavelength of 360 nm for CTX, measured at 291 nm for OCT.

### Cytotoxic activity and antiproliferative activity of the formulae

An *in vitro* cytotoxicity assay of all compounds was evaluated using the method described by Bakr *et al*.^59^. The study was performed in three human tumor cell lines: Michigan Cancer Foundation-7 (MCF-7), human hepatocellular carcinoma (HepG-2) and colon carcinoma (HCT-116). The cells were cultured with 10% fetal bovine serum in RPMI-1640 solution containing antibiotics (100 units/mL penicillin and 100 μg/mL streptomycin) and seeded in a 96-well plate at 1 × 10^4^ cell/well density and cultured at 37 °C, 5% CO_2_, for 48 hr. Cells were loaded with 6.25 and 100 μg/mL of blank ca-alginate beads, OCT only, CTX only, OCT alginate beads, CTX alginate beads and CTX-OCT-Alginate beads, and were incubated for 24 hr. The medium was discarded and cells were fixed in 150 μL/well 10% trichloroacetic acid (TCA) for 1 hr at 4 °C, and subsequently rinsed with water three times. Wells were stained with 70 μL/well 0.4% SRB for 10 min in the dark at 37 °C. The cells were then washed with 1% acetic acid to remove any unbound dye, then air-dried for 24 hr. The dye was solubilized in 50 μL/well 10 mM tris base (PH 7.4) for 5 min on a shaker at 1600 rpm. The optical density (OD) of each well was measured spectroscopically at 570 nm with an ELISA microplate reader (EXL 800 USA). The inhibitory concentration at 50% (IC_50_) was determined from an exponential curve of viability versus concentration of CTX and OCT. The viabilities were determined by the variable Sigmaplot software (systat software inc) as (A570 of samples/A570 of samples not being treated) × 100 and the amount IC_50_ (the concentration required to inhibit cell viability by 50 percent) for each compound. The data were collected and analysed to estimate the cell viability and growth effects of the testing compounds.

## Conclusions

Targeted drug delivery using specific drug carriers to treat tumors has many benefits including reduction of off-site targets and hence side effects, whilst also reducing the dose of the anticancer agent required for therapeutic response. We have successfully produced peptide–drug conjugates (PDCs) to function as potent drug delivery carriers due to their simplicity, versatility and relatively low cost for the construction of PDCs. In this study, CTX was successfully coated with OCT and loaded into alginate beads (CTX-OCT-Alg). FTIR studies confirmed the physical interaction of all components but found no interaction between CTX, OCT and alginate. The *in vitro* release results showed that CTX-OCT-Alg had a low drug release rate after 1 h in 0.1 HCl 1.2 pH, while in phosphate buffer pH 7.4, all drug contents were released over 5 h. Our results demonstrate CTX-OCT-Alg beads to have excellent gastro-resistant activity and efficiently deliver anti-cancer drugs to the higher pH environments of the colon with higher antiproliferative activity compared to free drug.
